# Beyond Malnutrition: The Role of Sanitation in Stunted Growth

**DOI:** 10.1289/ehp.122-A298

**Published:** 2014-11-01

**Authors:** Charles W. Schmidt

**Affiliations:** Charles W. Schmidt, MS, an award-winning science writer from Portland, ME, has written for *Discover Magazine*, S*cience*, and *Nature Medicine*.

Malnutrition in children can manifest in different ways; malnourished children can be underweight or obese, or their height can be stunted. Global health experts used to measure progress toward meeting childhood malnutrition goals on the basis of improvements in weight. But now stunting is the top priority. That’s because children who lose weight from a few days of being sick or hungry can readily gain it back, while the stunting that results from chronic malnourishment during early development has permanent consequences.^1^ More than merely a matter of appearance, stunting is a marker for an array of developmental problems, explains Reynaldo Martorell, a professor of international nutrition at Emory University. “The more stunted the child,” Martorell says, “the more likely it is that the brain, kidneys, and other organ systems will be affected.”

Studies have associated childhood stunting with IQ deficits, poor school performance, poverty, and higher risks for diabetes, heart disease, and stroke later in life.^1^^,^^2^^,^^3^^,^^4^ That’s both a health and an economic problem for affected countries, given how stunting impacts human capital and productivity.^5^ And although its prevalence is in steady decline—from 40% of children under age 5 in low-and middle-income countries in 1990 to 26% in 2011,^6^ mainly as a result of rising incomes and improved living standards—stunting remains pervasive. Worldwide, it’s estimated that 165 million children under age 5 are stunted, most of them in Africa and South and Central Asia.^6^ The number is expected to fall to 127 million by 2025, but that’s not enough to meet the goal of the World Health Organization (WHO) to reduce childhood stunting to 100 million cases by then.^7^

**Figure:**
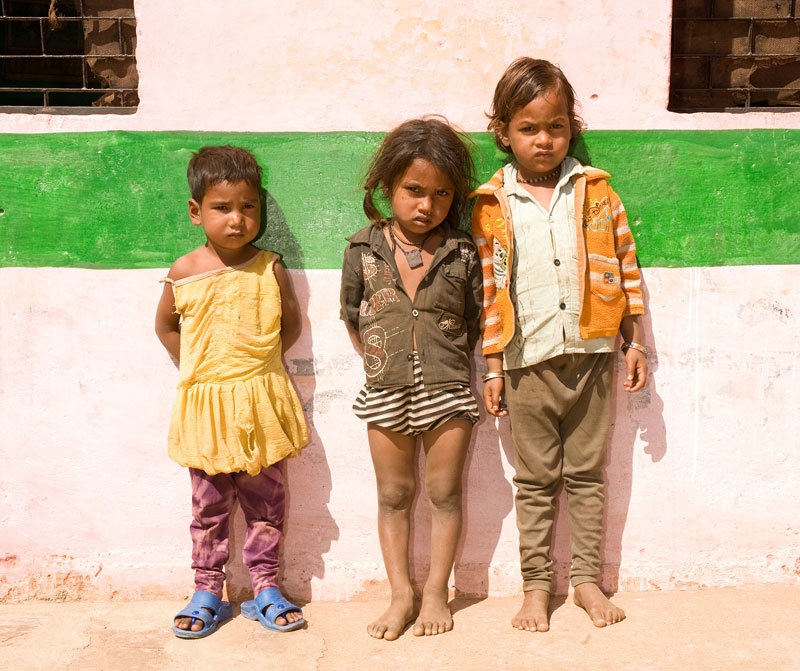
Worldwide, stunting affects an estimated 165 million children under the age of 5. Nutritional interventions are only part of the solution. © Roger Parkes/Alamy

It has become clear that nutritional interventions are only part of the solution to stunted growth. In countries such as India, for instance, stunting occurs even among well-fed children, and that’s led investigators to consider other causes, especially poor sanitation and hygiene. Evidence shows that children who live without adequate sanitation, hygiene, and clean drinking water don’t grow as well as children who do. Meanwhile, more than 626 million people in India (nearly half the population) routinely defecate on the ground outdoors,^8^ and this practice has been proposed^9^ as an important cause of India’s stunting epidemic, which affected an estimated 48% of the country’s children as of 2005–2006.^10^

In somewhat newer thinking, researchers are exploring the possibility that poor hygiene and a lack of sanitation induce a gut disorder called environmental enteropathy (EE) that diverts energy from growth toward an ongoing fight against subclinical infection. Nutritionists are now collaborating with experts in a field known as water, sanitation, and hygiene (WASH), and their combined efforts are helping to galvanize regional programs to improve hygiene in countries afflicted with high stunting rates.

In November 2014, for instance, experts will convene in New Delhi, India, for a regional conference titled Stop Stunting: Improving Child Feeding, Women’s Nutrition, and Household Sanitation in South Asia. India’s new prime minister, Narendra Modi, has also spearheaded a campaign known as Swachh Bharat (Clean India)^11^ in part to eliminate open defecation in the country by 2019.^12^

## The Historical View

Stunting has been observed for centuries, but it wasn’t until 1973 that the current standard definition for the term was introduced by the nutritionist John Waterlow, from the London School of Hygiene and Tropical Medicine. Writing in *The Lancet*, Waterlow distinguished between deficits in height-for-age, which he called stunting, and deficits in weight-for-height, or wasting.^13^ He also described wasting as “a fairly acute state of malnutrition,” whereas stunting was said to result from undernutrition over a long period, causing a “retardation in linear growth.”^13^

Waterlow quantified stunting in terms of standard deviations below the expected height for a given age. Today this metric is known as the height-for-age *z* (HAZ) score; stunted children are said to be at least 2 standard deviations below the age- and sex-specific median height as determined by the World Health Organization (WHO) Child Growth Standards.^14^

**Figure:**
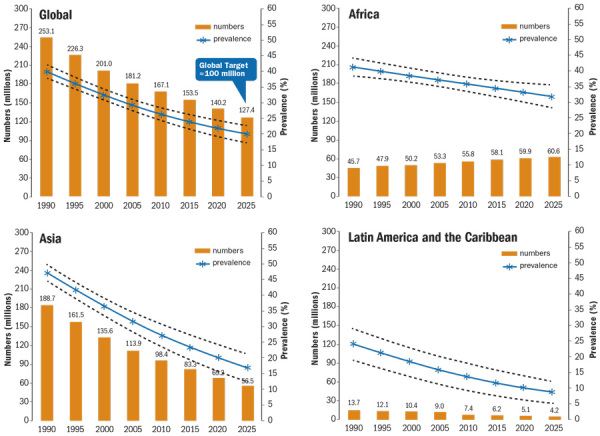
Trends in child stunting by UN region (1990–2025) Source: de Onis et al. (2013)^7^

These standards were developed using reference data collected through the Multicentre Growth Reference Study (MGRS), which the WHO conducted for nearly six years in six locations: Davis, California; Muscat, Oman; Oslo, Norway; Pelotas, Brazil; Accra, Ghana; and South Delhi, India. Growth measures were taken weekly during infancy, declining to monthly and then bimonthly as the children grew older to account for the especially rapid growth that occurs in babies. And importantly, measurements were made only of healthy, well-nourished, breastfed children from middle-class backgrounds, whose growth rates were assumed to be optimal and uncompromised by the effects of poverty.^15^

MGRS results published in 2006 produced a striking finding: Infants and young children follow similar growth patterns regardless of race or ethnicity.^16^ Maureen Black, director of the Division of Growth and Nutrition at the University of Maryland School of Medicine, explains that genetic differences that render some Arctic peoples short, for instance, and the Maasai of Kenya tall are expressed later in life. Black says that 97% of healthy young children should be of a height that falls within the HAZ’s statistical boundary. “So theoretically, just 3% of the child population should be less than two standard deviations shorter than the [median] HAZ,” she says. “But we see values much higher than that—in Bangladesh, for instance, nearly half the kids are stunted.”^17^

## Beyond Nutrition

Nutritionists have tried dozens of approaches to prevent stunting, such as micronutrient supplements for pregnant women and children (especially growth promoters including iron, zinc, calcium, and folate); increased availability of fat-fortified commercial products such Nutributter and Plumpy’nut; a concerted push to encourage breastfeeding during the first six months of life; and efforts to improve the nutritional quality of the complementary foods babies eat while weaning.^6^

But Jean Humphrey, a professor of human nutrition at the Johns Hopkins Bloomberg School of Public Health, says none of these interventions has been able to eliminate stunting completely. At best, she says, they improve growth by about a third of the typical height deficit in stunted Asian and African children. “This tells us that dietary improvements are important but not sufficient,” she says. “If we really want to eliminate stunting, we need to do more.”

Meanwhile, mounting evidence has shown that poor hygiene and sanitation also constrain linear growth in children. One study found that Bangladeshi children who had access to clean drinking water, improved toilets, and facilities for handwashing with soap, for instance, had a roughly 50% improvement in HAZ scores compared with control children who didn’t.^18^ Similar results emerged from studies in Sudan^19^ and Mexico,^20^ yet it was unclear exactly why poor WASH would contribute to stunting and WASH improvements would help to ameliorate it.

**Figure:**
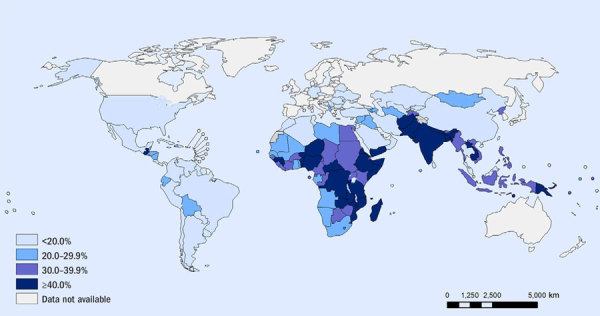
Stunting among children under age 5 years Source: de Onis et al. (2013)^7^

Scientists at first hypothesized that children who live with poor hygiene become stunted because they have chronic diarrhea. A 2008 meta-analysis that pooled results from nine studies supported that hypothesis; the authors revealed a strong association between impaired linear growth and five or more episodes of diarrhea within the first two years of life.^21^ But in other studies, the evidence was less compelling. Researchers in Karachi, Pakistan, for example, found that handwashing by children and their parents was highly protective against diarrhea but had no effect on stunting.^22^

Investigations into potential links between diarrhea and stunting are ongoing. Meanwhile, researchers have increasingly turned their attention to the condition known as EE. This illness was first documented nearly 50 years ago, after military veterans and Peace Corps volunteers started coming home from South and Southeast Asia with an asymptomatic intestinal condition that looked like a mild form of celiac disease. Then known as tropical enteropathy, the disorder was seen initially on biopsy—intestinal linings tended to thin out and fill with inflammatory secretions. Experts describe people with EE as having “leaky guts,” and the disorder was eventually found to be pervasive in the developing world. Interestingly, EE generally resolves in people who relocate or return to developed countries.^23^

## Exploring EE

EE isn’t easy to diagnose; biopsies are definitive but impractical for research, says Stephen Luby, a professor at Stanford University’s Freeman Spogli Institute for International Studies. Scientists generally rely on indirect approaches for diagnosing EE, the most common being a test of what’s called the lactulose:mannitol (L:M) ratio. To administer the test, which is also used to diagnose Crohn’s disease, scientists give children a drink that contains the two sugars lactulose and mannitol. Mannitol is a small sugar that is readily absorbed through the small intestine, while lactulose is a much larger sugar that is only partly absorbed. If the child’s intestine is abnormally permeable (“leaky”), then unusually large amounts of lactulose pass into the blood and are eventually excreted in urine. High lactulose levels in urine therefore predict EE, “but the test is hard to standardize and interpret,” Luby says. “Stunting is easy to measure; EE, not so much.”

Despite its limitations, the L:M ratio has provided much of the evidence in support of a role for EE in stunting. The most highly cited of these studies was published in 1991 by researchers who concluded that abnormal L:M ratios could explain nearly half the impaired growth observed in a group of Gambian children.^24^ Humphrey says the Gambian study was critical because it was the first to show that interventions to reduce clinical diarrhea could be highly successful without changing the incidence of stunting at all. In a followup study, the same research team linked impaired growth in Gambian infants to intestinal enteropathy and high levels of plasma immunoglobulins and endotoxin antibodies.^25^

**Figure:**
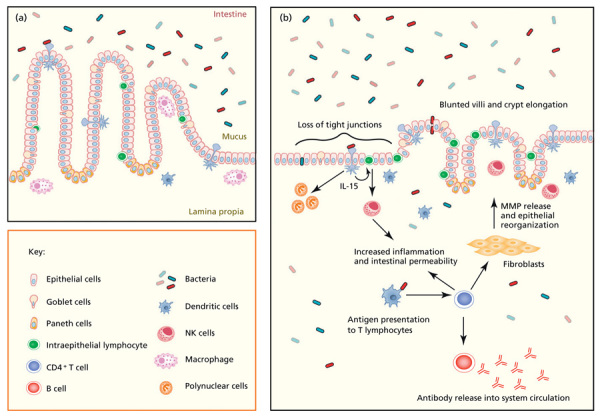
**Stunting among children under age 5 years** (a) In normal intestines, goblet cells secrete a layer of mucus that protects epithelial cells from exposure to bacteria. Macrophages protect against bacteria entering the lamina propria while minimizing tissue injury. Tight junctions are undisrupted. Villi are long and finger-like, separated by relatively shallow crypts. (b) Recurrent gastrointestinal infection results in disruption of the mucus layer and loss of barrier function. Luminal bacteria bind to dendritic cells and intraepithelial lymphocytes (IELs) on the epithelial surface. The immune response engages, and the intestine becomes more permeable as a result of IEL activation. Increased pathogenic infiltration into the lamina propria results in a cycle of inflammation that ultimately changes the architecture of the intestine, with increased IELs and lymphocytic infiltration in the lamina propria, as well as villus atrophy and crypt hyperplasia. Figure reprinted from Korpe and Petrie (2012).^30^ Caption adapted by EHP.

According to Humphrey, ingested microbes, once they pass through a leaky gut into the blood stream, set off a continual low-level immune reaction. She says this reaction drains off energy and nutrients that children would otherwise need to grow to their full potential. “The immune response in EE is lifesaving, but it’s also metabolically expensive,” she says. Humphrey now believes that diarrhea is a minor contributor to stunting when compared with EE, which she calls a “chronic immune-activating disorder that chips away at growth and affects kids on a population level.”

Luby points out that in extreme cases, chronic diarrhea and EE might go hand in hand, making it difficult to tease out their individual effects. But he adds that a number of different organisms can infect the intestinal tract and cause gut dysfunction without inducing diarrhea. Specific causative bacterial agents in EE remain unknown, Luby says, but preliminary investigations in Bangladesh found that children with access to better hygiene had fewer intestinal pathogens, lower L:M ratios, less immune stimulation, and better linear growth.^18^

Two major randomized studies, each funded by the Bill and Melinda Gates Foundation, are now investigating the potential role of EE in childhood stunting. Humphrey leads one of them—the Sanitation, Hygiene, Infant Nutrition Efficacy (SHINE) Project^26^—which is under way in Zimbabwe. The study is comparing growth outcomes in relation to measures of gut function among villagers at two locations. One of the villages has access to latrines, treated drinking water, and programs to limit exposure to fecal microbes, while the other does not. The second study, known as WASH Benefits, is co-directed by Luby. It is now under way at multiple sites in Bangladesh and Kenya.^27^ Like SHINE, the WASH Benefits study will measure EE markers in relation to stunting, among many other end points. Villagers in both Bangladesh and Kenya have been randomized to a variety of interventions involving water quality, sanitation, handwashing, and nutrition.

## Cultural Barriers

Luby says subjects in the WASH Benefits study have been highly cooperative. The team had extensive anthropological support, he says, and “when walking into villages during unannounced visits we see chlorine in drinking water, soap in the washing stations; all our indicators are way above benchmark.” Yet Luby concedes that the cooperation seen in research settings might not carry through in a real-world intervention. When it comes to fighting stunting, he says, it’s easier for health groups to dispense micronutrient tablets than it is to change an entire community’s drinking water, sanitation, and handwashing behavior.

Other researchers echo similar views. Francis Ngure, an independent consultant and researcher at Cornell University, found that even though soils ingested by infants and toddlers crawling on dirt floors in rural Peru were often highly contaminated with chicken feces and fecal bacteria, villagers generally were unable or unwilling to take steps to limit their exposure. According to Ngure, villagers more often said they couldn’t afford to keep poultry in cages and that they preferred the taste of free-range chicken meat and eggs. Similarly, confining children to clean play spaces elicited negative reactions from villagers in Zimbabwe, who felt they interfered with cultural concepts of play.^22^

Sue Coates, chief of the WASH Section with the United Nations Children’s Fund India, adds that when it comes to stopping open defecation in India, toilet construction isn’t the main problem. “The real problem is promoting a social demand for initial and sustained toilet use,” she says. It would be like telling Americans or Europeans they should now defecate in the street, she explains. “We’re overlaying our belief system, norms, and mindsets on others, and this is a very complex development scenario to tackle,” Coates says.

For the Indian government’s new Swachh Bharat campaign, which launched in October 2014, frontline health workers are trying to associate toilet use with dignity, especially for women and girls.^28^ UNICEF and other groups are also urging a link between infant and child care and the need for mothers to wash their hands with soap before engaging in breastfeeding and complementary feeding by equating hand hygiene with better nutrition and child growth.^29^

Maintaining this trend is both a health and an economic imperative for developing countries. Stunted children will likely never reach their full potential as productive members of their own societies, emphasizes Mercedes de Onis, coordinator of the Growth Assessment and Surveillance Unit in the WHO Department of Nutrition. “But stunting isn’t something you can address with a silver bullet,” she says. “It has divergent components, and what causes it in one location may not be what causes it in another. There’s a lot we can do, but ultimately stunting is tied to poverty. And eradicating poverty isn’t easy.”
